# Is it time to assign *Lactiplantibacillus plantarum* as a keystone species in the *Drosophila melanogaster* microbiota?

**DOI:** 10.1128/aem.01491-25

**Published:** 2025-09-25

**Authors:** John M. Chaston

**Affiliations:** 1 Brigham Young University 6756, Provo, Utah, USA; Michigan State University, East Lansing, Michigan, USA

**Keywords:** keystone species, core microbiota, priority effects, gut acidification

## Abstract

Recent work by Yang et al. published in *Applied and Environmental Microbiology* (91:e00707-25, 2025, https://doi.org/10.1128/aem.00707-25) offers fresh insights into the important roles played by *Lactiplantibacillus plantarum* within the microbial community of the fruit fly *Drosophila melanogaster*. This commentary summarizes their findings in the context of earlier studies, highlighting the unique and influential position of *L. plantarum* in shaping the biology and microbial dynamics of *D. melanogaster*.

## COMMENTARY

A growing body of research highlights the prominent role of *Lactiplantibacillus plantarum* (*Lp*) in the microbiota of *Drosophila melanogaster* (*Dm*), demonstrating its capacity to influence host traits and microbial community composition. *Lp* has been shown to influence host nutritional indices (1, 2), life history traits (3–8), memory (9), sleep (10), feeding behavior (11), gut pH and motility (12, 13), gene expression (14), and resistance to insecticides (15), fungal infection (16, 17), and bacterial infection (18–20). However, not all of *Lp’*s actions are necessarily beneficial to its insect host (21–23). Perhaps the strongest evidence of *Lp*’s unique status among *Dm*-associated microbes is its frequent mention in the literature. A PubMed search yields 107 citations containing both “Drosophila” and “plantarum” (Table 1), a number that, while smaller than the 322 matches for “*Drosophila*” and the pathogen “*Pseudomonas*,” still accounts for more than half of the 197 abstracts referencing common *Dm*-associated lactic acid bacteria (LAB) under both old and new LAB nomenclature. This also represents at least a 50% increase in citations compared to other commonly studied bacterial strains in *Dm* research. Broader literature searches are unlikely to change the central observation: *Lp* stands out as a member of the *Dm* microbiota. Recent work by Yang et al. (24) reinforces this view by showing that *Lp* reshapes the colonization landscape in *Dm*, that priority effects influence microbiota composition and host outcomes, and that species-specific microbial impacts are critical considerations.

**TABLE 1:** PubMed search terms and results

Search term	No. of results for:	
	Drosophila AND	16S Drosophila AND
fungus	8,511	n.s.^*a*^
bacteria	8,140	n.s.
pathogen	4,055	n.s.
virus	3,631	n.s.
microbiome	773	n.s.
microbiota	648	n.s.
pseudomonas	322	8
(limo*bacillus OR levi*bacillus OR lact*bacillus)	197	26
lactobacillus	164	24
acetobacter	116	24
plantarum	107	9
tropicalis	64	2
pomorum	22	3
brevis	17	2
shigella	13	1
leuconostoc	10	3
pasteurianus	6	1
fructivorans	3	1
pasteurella	2	0
thailandicus	2	0

^*a*^ n.s., not searched.

## *Lp* RESHAPES THE *Dm* COLONIZATION LANDSCAPE

Yang et al. demonstrate that *Lp* acidifies multiple regions of the *Dm* gut, including the foregut, hindgut, and rectum, while simultaneously excluding the pathogen *Serratia marcescens*. They also found that the low pH in the copper cell region occurs independently of bacterial colonization, contrasting with earlier findings that linked this acidification to *Lp* (12). These results underscore one mechanism by which *Lp* can shape the gut environment, potentially contributing to protection against bacterial (18, 20) or fungal infections (16, 17), as well as resistance to insecticides (15). However, *Lp*’s influence extends beyond acidification. *Lp* has also been associated with changes in gut motility (13), production of bacteriocins (25), metabolic cross-feeding with other microbes (26–28), and modulation of immune gene expression (14). The host has even evolved specific antimicrobial responses to *Lp* (29). Moreover, *Lp* can increase *Dm* susceptibility to the insecticide chlorpyrifos by metabolizing this compound to the more toxic chlorpyrifos oxon, a process that is unlikely to occur only in response to decreased pH (21). These findings collectively highlight that *Lp*’s impact on the gut environment involves a diverse array of mechanisms beyond acid production.

Beyond its influence on the composition of the microbial community, *Lp* functions as an ecosystem engineer within *Dm*, inducing both morphological and physiological changes in host tissues (30). Flies colonized with certain *Lp* strains have a larger lumenal space in their proventriculus and stain positively for wheat-germ agglutinin, a host-associated molecular pattern detected in the same areas where microorganisms colonize many invertebrate hosts (31–34). These influences facilitate colonization by *Acetobacter* species, which are prevalent constituents of the *Dm* gut microbiota. Collectively, these findings emphasize the pivotal role of *Lp* in shaping a gut environment that either promotes or inhibits colonization by other microbial taxa. They also show that while most *Lp*-mediated effects are associated with beneficial microbial communities and host phenotypes, some influences may diverge from this trend, indicating a nuanced and strain-specific impact on host–microbe interactions.

## PRIORITY EFFECTS DETERMINE *Dm* MICROBIOTA COMPOSITION AND HOST INFLUENCES

*Lp*-dependent modifications to the structure and function of the gut also support the role of *Lp* as a priority colonizer in *Dm*. Priority effects describe how the order of colonization can determine colonization outcomes, and are a feature of many host-associated microbial communities (35). Early colonization by *Lp* has been shown to increase the bacterial loads of certain co-colonizing species (30). However, in separate experiments involving a different *Lp* strain and separate rearing conditions, no consistent increase in *Acetobacter* loads was observed (2), suggesting priority effects may be strain-specific and context-dependent, particularly with respect to environmental factors, such as diet. If the effects are not strain specific, these findings indicate that microbial load in adult flies is an unreliable proxy for priority effects and may not represent their primary outcome.

Beyond microbiota composition, Yang et al. convincingly show that priority colonization by *Lp* also determines a key fly life history trait—lifespan. Flies pre-colonized with *Lp* and then exposed to the oral pathogen *S. marcescens* exhibited a mean lifespan of 27 days, compared to 17 days for naïve flies exposed to *S. marcescens*, and 37 days for *Lp-*colonized flies not treated with the oral pathogen. In contrast, the median lifespan of flies co-colonized (inoculated at the same time) with *Lp* and *S. marcescens* was 15 days, showing that the lifespan protection was a true priority effect, attributed to acidification of the gut. This finding raises questions about whether pre-colonization with *Lp* can enhance colonization proficiency or loads of other microbiota members in conditions where simultaneous co-colonization fails to do so (2).

Similar experimental approaches, including pre-colonization, pulse-chase, and bacterial tracking approaches, have been employed by other colleagues of Yang et al. to investigate priority effects in flies colonized with laboratory or wild microbial strains. Different priority effects have been observed between *Lp* strains, for example, as an *Lp* strain with high, but not low, colonization proficiency was able to exclude both heterologous and isogenic strains from its established niche (36). This exclusion was visibly confirmed in the proventriculus using fluorescently labeled *Lp* strains with differing colonization efficiencies (30). The fine-scale temporal resolution, large sample sizes, analysis of individual animals, and imaging of live flies all emphasize the utility of *Dm* as a model system to dissect priority effects in microbial colonization and their downstream impacts on host traits, behavior, and physiology.

## SPECIES-SPECIFIC INFLUENCES ARE A MAJOR CONSIDERATION

Many of the influences exerted by *Lp* on *Dm* and its microbiota, including those mediated through priority effects, are strain-specific. Only select *Lp* strains have been shown to remodel the host’s proventriculus (30), rescue developmental delays associated with undernutrition (37, 38), and colonize the host with high efficiency (36, 39). In contrast, some traits appear to be conserved across *Lp* strains. For instance, in a study where gut acidification was microbiota-responsive, it was similarly influenced by both a fly isolate and a human saliva isolate of *Lp* (12), suggesting that this function may represent a broad *Lp*-facilitated community trait.

Despite these insights, relatively few studies have systematically evaluated whether such influences are species-specific or conserved across broader taxonomic levels. The prevailing assumption is that many of these effects are unique to *Lp*. For example, the unique cell wall structure of *Lp* likely contributes to its specific effects on host immunity and developmental timing, distinguishing it from other LAB (5, 8, 40). Dozens of manuscripts have compared phenotypic influences of several bacterial strains or species on *Dm*, often demonstrating that observed effects are specific to *Lp* relative to a narrow panel of strains. Broader surveys encompassing dozens of bacterial species, including *Lp*, have revealed substantial variation in traits, such as persistent colonization, and hosts' triacylglyceride content, development rate, starvation resistance, and lifespan (4, 41–43). Notably, in each of these studies, *Dm* phenotypes did not differ between colonization with fly- or human-derived *Lp* isolates, reinforcing the notion that many *Lp*-mediated effects may be shared between *Lp* strains.

It will be unrealistic to expect most or any of these traits to be comprehensively and saturatingly defined as exclusively species- or strain-specific to *Lp*. However, these findings together show that some *Lp*-dependent traits can be classified as specific or general to *Lp* and its relatives at high and low taxonomic resolution.

## WHY *Lp*?

Several factors may explain the disproportionate focus on *Lp* in studies of the *Dm* microbiota relative to other microbial taxa. One plausible explanation is its status as a well-characterized organism, which has led to its frequent inclusion in subsequent analyses. However, *Lp* may also merit this attention due to specific biological and experimental attributes that distinguish it from other microbiota members.

A primary factor is the culturability of *Lp*. Numerous studies have examined *Lp* based on its consistent recovery from fly stocks (2, 20, 37, 44–47). *Lp* typically forms large, yellow colonies that are visually distinct from the off-white, morphologically similar colonies produced by other fly-associated LAB, such as *Lactobacillus*, *Levilactobacillus*, *Weissella*, and *Leuconostoc* (data not shown [48]). This distinctive morphology and ready culturability likely contribute to preferential selection and study of *Lp*.

A second factor is *Lp*’s prevalence in fly populations. *Lp* is frequently reported in studies of the *Dm* microbiota (21, 45, 49–56), although not all studies report their findings to species-level resolution. In a targeted literature search incorporating the term “16S” to the search parameters used in the literature search above, *Lp* appeared more frequently than any other microorganism queried (Table 1). Assuming a relative balance of wild and laboratory studies of the microbiota, this observation contrasts with suggestions that LAB may be more prominent in laboratory than the wild fly microbiota (57, 58). However, recent analyses suggest that technical limitations in wild fly sampling, such as the use of broadly distributed baits like apples, may underrepresent LAB diversity (59). To further assess *Lp* prevalence, I examined taxonomic assignment files from eighteen 16S rRNA sequencing studies previously analyzed in my lab (targeting V1-2 and V4 regions), all using the Greengenes database. Reads assigned to *Lp* were present in four of six laboratory fly samplings, one of six wild fly samplings, and one of five samplings involving flies reared on laboratory diets in the wild. In contrast, *Levilactobacillus brevis* was detected in every data set, including the two that questioned the relevance of LAB in wild flies (data not shown [57], supporting data set S1 [58]). These findings suggest that while *Lp* is highly prevalent, it is not the most ubiquitous LAB across all sampling contexts. Limitations of this analysis include a lack of sample-level resolution, poor species-level discrimination of *Acetobacter* in 16S data (with *Acetobacter aceti* being the only species-resolved taxon in Table 2), and geographic bias toward Utah and the eastern United States.

**TABLE 2:** Bacterial presence in 16S marker gene data sets analyzed in the Chaston lab

Citation	*Lp* present?	*L. brevis* present?	*A. aceti* present?	Environment	Location	Year samples collected or sequenced
(60)	Yes	Yes	Yes	Lab	AZ	2019
Unpublished data	Yes	Yes	Yes	Lab	AZ	2019
Unpublished data	No	Yes	No	Lab	AZ	2020
Unpublished data	Yes	Yes	Yes	Lab	Utah	2022
Unpublished data	Yes	Yes	Yes	Lab	Utah	2022
Unpublished data	No	Yes	No	Lab	Utah	2023
Unpublished data	No	Yes	No	Wild on lab diet	PA	2014
Unpublished data	No	Yes	No	Wild on lab diet	PA	2016
Unpublished data	No	Yes	No	Wild on lab diet	PA	2017
Unpublished data	Yes	Yes	Yes	Wild on lab diet	PA	2022
Unpublished data	No	Yes	No	Wild on lab diet	NH	2023
(61)	No	Yes	No	Wild	Utah	1991–1993
(61)	No	Yes	No	Wild	East coast, USA	2009
(57)	No	Yes	Yes	Wild	Yale	2018
(59)	Yes	Yes	Yes	Wild	Utah	2020
(59)	No	Yes	Yes	Wild	East coast, USA	2021
(59)	No	Yes	No	Wild	Utah	2022

Finally, *Lp* has garnered attention due to its biologically interesting phenotypes in *Dm*. As discussed above, *Lp* influences host physiology and microbial community structure in strain- and species-specific ways. Several studies have focused on *Lp* only after screening other microbiota members and identifying its unique effects (23, 36, 37). These findings suggest *Lp* is not only experimentally tractable and prevalent, but also biologically compelling.

## *Lp* AS A KEYSTONE SPECIES OF THE *Dm* MICROBIOTA

Interest in identifying keystone and core taxa in microbiomes has varied over time, complicated by technical challenges and ongoing debates regarding definitions and criteria (62–66). A central aim of such efforts has been to isolate key microbial members whose roles may illuminate the dynamics of complex communities that are otherwise difficult to study in their entirety.

The concept of keystone species originated in studies of megafaunal ecosystems, initially centered on predation, and has since evolved to encompass a range of ecological functions (66–69). Criteria for keystone designation have included disproportionate influence relative to abundance, exclusion of invasive species, facilitation of other community members, ecosystem modification, and classification as dominant, foundational, or demonstrably important taxa (69). In microbial ecology, recent efforts have focused on computational approaches to identify keystone species from sequencing data, which are often more readily available than functional data sets (63). Co-occurrence analyses are a commonly employed method, though it has been recognized that computational inference alone is insufficient for keystone designation without experimental validation (64, 65). Consequently, definitions and methodologies for identifying keystone taxa remain diverse and context-dependent.

*Lp* appears to fulfill several classical criteria for keystone status in *Dm*. It functions as an environmental architect by reshaping the environmental conditions of the fly gut and cross-feeding with other community members. For example, Yang et al. showed that *Lp*-mediated pH remodeling directly influences microbiota composition *in* the fly gut, independent of dietary effects (24). However, co-occurrence analyses of the *Dm* microbiota (which are few) have not consistently identified *Lp* as a highly connected taxon (52, 70). Also, *Lp* was not detected in connectivity analyses of the *Drosophila suzukii* microbiota (71, 72). These findings suggest that while *Lp* may qualify as a keystone species under classical ecological definitions, it may not be readily identified through popular computational metrics. This discrepancy underscores the limitations of co-occurrence analyses in *Dm* microbiota research and supports the candidacy of *Lp* as a keystone species.

In contrast, the designation of *Lp* as a core member of the microbiota is less straightforward. The Human Microbiome Project originally aimed to define a core human microbiota, though early definitions varied widely (73). Core taxa have been defined based on universality, abundance, or prevalence thresholds, often clustered at taxonomic or functional levels. As with keystone taxa, these definitions are susceptible to manipulation to fit specific organisms. This is particularly relevant in *Dm* and other animals where the microbiota is highly variable and lacks evidence of host mechanisms for consistent microbial transmission across generations (52, 74). As reported above, while *Lp* is common in laboratory flies, it is rare or absent in wild populations. In contrast, *L. brevis* appears to be a more consistent and prevalent member of the *Dm* microbiota, making it a stronger candidate for core status.

The strain-specific nature of *Lp’s* effects further complicates its classification. If only certain *Lp* strains perform keystone functions, it raises questions about the appropriate taxonomic resolution—species, subspecies, or strain—for defining core or keystone taxa. For instance, if some *Lp* strains lack the functional traits necessary for gut colonization or environmental remodeling, a species-level designation may be inappropriate. Thus, while *Lp* may be considered a core member of laboratory fly microbiota, its absence in wild populations and variability across strains suggest that it may be a stretch to assign it “core” status.

This ambiguity prompts further questions. Do flies that lack *Lp* also lack acidification-based protection against intestinal pathogens? Are microbial taxa that rely on *Lp*-mediated priority colonization at risk of failing to establish in these hosts? Do other community members compensate by performing analogous functions, or are key traits, such as plasmid-borne adhesin islands distributed across diverse taxa, obscuring taxonomic signals (39)? Moreover, given the high prevalence of *L. brevis*, are there overlooked functional roles that merit further investigation? Alternatively, has *L. brevis* received less attention due to historical or methodological biases? These all seem like fruitful areas for future research in the *Dm* microbiota.

In sum, *Lp* occupies a distinctive position within the *Dm* microbiota. It has been the subject of more studies than any other microbial taxon in this system, likely due to its culturability, prevalence in laboratory settings, and broad influence on host physiology, immunity, and nutrition. The roles of *Lp,* as highlighted by Yang et al., including changing the gut environment and where priority effects have downstream influences on host life history, emphasize the importance of *Lp* as a *Dm* community member. Thus, while definitions of keystone and core taxa remain fluid, the body of work by Yang and others supports the classification of *Lp* as a keystone species in the *Dm* microbiota.
